# Toll-like receptor 7 and RIG-I-like receptors expression in peripheral blood mononuclear cells of naïve patients with hepatitis C

**DOI:** 10.1186/s13104-023-06626-2

**Published:** 2023-11-22

**Authors:** Atena Gilanipour, Ali Teimoori, Seyed Alimohammad Arabzadeh, Hamid Reza Mollaie, Elham Mousavi

**Affiliations:** 1 Department of Medical Microbiology (Bacteriology and Virology), Afzalipour Faculty of Medicine, Kerman University of Medical Sciences, Kerman, Iran; 2 Department of Virology, Faculty of Medicine, Hamadan University of Medical Sciences, Hamadan, Iran; 3 Medical Mycology and Bacteriology Research Center, Kerman University of Medical Sciences, Kerman, Iran

**Keywords:** Hepatitis C virus, Toll-like receptor7, RIG-I-like receptors

## Abstract

**Background:**

The proper function of Pattern Recognition Receptors (PRRs) as a part of the host immune system can eliminate numerous pathogens from the body. However, some viruses can manipulate PRRs to escape the innate immune system. As there is controversy in the activation of PRRs in patients infected with HCV, we decided to evaluate the gene expression changes of PRRs in HCV cases compared to the healthy control.

**Methods:**

In this study, the relative expression of Toll-like receptor 7, RIG-I, and MAD-5 in peripheral mononuclear blood cells of twenty HCV patients and twenty healthy controls of the same gender and age were analyzed by quantitative Real-time PCR.

**Results:**

Our results showed that the expression of RIG-I and MAD-5 significantly increased in HCV-infected samples compared to the controls (P value:0.01; P value:0.05), while the expression of TLR7 was similar between the case and the control group (P value:0.1).

**Conclusion:**

It seems in suppressing HCV, RIG-I and MAD-5 receptors are likely to be more activated than TRL7 in HCV patients. The lack of TLR7 gene expression might reflect the defect of the host in the stimulation of the innate immune system through the TLR7 pathway.

## Introduction

The Hepatitis C virus (HCV) is considered as one of the major causes of hepatitis, liver cirrhosis and hepatocellular carcinoma [1]. Although the innate immune system has an important role in clearing HCV from the body, nearly 40% of individuals are not able to spontaneously resolve the infection [2]. Despite many efforts, the reason for the persistence of HCV in some people has not been fully understood. Among Pattern Recognition Receptors (PRRs), Toll-like receptors(TLRs) (such as TLR3,8 and 7) and Retinoic acid-inducible gene I (RIG-I)-like receptors (RLRs) including RIG-I and MAD-5 recognize the virus genetic material (ss RNA or ds RNA) in the initial phase of infection and lead to stimulation of INFs (Interferons) signaling pathway [3, 4]. INFs elevate the expression of interferon-stimulated genes (ISGs) and their products block every step of viral replication by applying a variety of mechanisms [5]. To exemplify, The OAS (oligoadenylate synthetase) proteins are one of the ISG products that are produced in HCV-infected cells. As a matter of fact, the OAS activates latent cellular RNase L, which efficiently degrades viral genomes in HCV-infected cells [6, 7]. Therefore, the failure in function of innate receptors can cause an inappropriate viral response. In several clinical studies, the expression level of a variety of PRRs in HCV positive patients have been evaluated [8–10]. Among findings obtained from different studies, significant differences are observed. To exemplify, the study by Dolganiuc and Dehghan et al. suggested an increase in mRNA expression levels of TLRs during HCV infection [8, 9], while Atencia and Motavaf et al. showed that TLR3 and TLR7 mRNA levels are significantly downregulated in patients with HCV infection compared to healthy controls [10, 11]. Recent scientific evidence proposes some HCV proteins suppress the TLR-dependent signaling pathway through alteration of TLRs expression levels [12, 13]. It seems that HCV targets TLR-mediated signals to modulate the host immune system in order to persist in the host [11, 14]. However, some other studies believe the lack of spontaneous clearance of HCV in patients depends on host genetics factors [15].

Recently, TLR agonists have been introduced as one of the potential agents for treating infections [16]. For instance, Isatoribine, an agonist of TLR7, showed a significant effective in reducing the viral load in the blood of hepatitis patients [17]. In another study, TLR agonists was proposed as a viable strategy for preventing respiratory viral diseases [18].

Although, those chemical molecules might be a promising approach for treating some viral infections, in regard HCV infection, prescription of PRRs stimulators might be ineffective because in some HCV patients, the expression of some PRRs are suppressed. As mentioned above, a significant discrepancy was found in expressing of PRRs genes among HCV patients. Therfore, to determine the ability of the host immune system to fight off HCV infection, we decided to examine TLR7, RIG-I, and MAD-5 expression on peripheral blood mononuclear cells (PBMCs) in HCV-infected patients who did not receive any therapy compared to normal controls. The results of this study can show us a better understanding of the TLR response during HCV infection which can be beneficial for the development of new therapeutic approaches against HCV infection.

## Patients and methods

### Patients

A case–control study was conducted on two groups of HCV patients and healthy people. In patient group, twenty new cases of patients with HCV infection were included. To avoid any interfering variables, people with any viral infection, autoimmune diseases and alcoholic liver disease were excluded. Anti-HCV antibody was detected by enzyme-linked immunosorbent assay (ELISA) in serum of all patients. Moreover, the real time PCR method was applied by the reverse transcription-polymerase chain reaction (RT-PCR) kit (Roche Applied Sciences, RAS, Mannheim, Germany) to confirm the presence of HCV RNA in the blood. To exclude patients with fibrosis, a gastroenterologist was examined all patients. For healthy subjects, twenty people who were age and sex matched with patients, were selected as controls. The Ethics Committee of Kerman University of Medical Sciences, Kerman, Iran approved this study with the code IR.KMU.REC.1398.181. All subjects agreed to participate in the study by signing a written informed consent.

## Real time PCR

### RNA extraction and reverse transcription

To isolate PBMCs, five ml venous blood samples were collected in tubes containing EDTA and immediately the PBMCs were separated by centrifugation on a Ficoll gradient (FicoLEX, France). Subsequently, total cellular RNA was extracted using Trizol reagent (YTzol Pure RNA, Iran).The quantity and purity of RNA were determined by measuring the absorbance at 260 nm and the A260/A280 ratio in a UV spectrophotometer (Nanodrop Inc, Wilmington, DE USA). The same amount of RNA from the samples was used to perform reverse transcription using the QuantiTect Reverse Transcription Kit (QIAGEN, Germany) according to the manufacturer's recommendations.

### Real-time polymerase chain reaction

The primers for TLR7, RIG-I, MAD-5 and HPRT genes were designed and validated in Primer BLAST, Beacon Designer and Primer Quest™ Tool respectively (Table1). The real-time PCR was performed using the Real Q-PCR Master Mix kit (Amplicon III, Denmark) according to the kit protocol. Briefly, 1 μL of cDNA was used for each PCR with 0.5 uM of forward and reverse primers in a total volume of 10 μL. All amplifications were done in QIAGEN's real-time PCR cycler**.** The thermal cycling conditions comprised of 10 min at 95 °C, followed by 45 cycles at 95 °C for 15 s, 58 °C for 30 s, and 72 °C for 15 s. To control for specificity of the amplification products, after 45 cycles a melting curve was generated in the range of 55 °C to 95 °C. Hypoxanthine Guanine Phosphoribosyl transferase (HPRT)was used as a housekeeping gene for normalization of the amount of mRNA expression of the target gene. In the following, the comparative ∆∆Ct method was used to measure the relative expression of each gene as mentioned below. At first, all samples were normalized with calculation of ∆Ct = (ct,target gene)–(ct, housekeeping gene). Then, the difference between the mean value of ∆Ct of patients group from the mean value of ∆Ct of healthy group was calculated as ∆∆Ct. Eventually, fold increase in the expression of specific mRNA in HCV patients compared to normal control was calculated as 2^−(∆∆Ct)^.

**Table1:** Primer sequence

Gene	Forward	Reverse
HPRT	TAGCCCTCTGTGTGCTCAAG	ATTACTTTTATGTCCCCTGTTGAC
TLR7	TCAAGAAAGTTGATGCTATTGGG	CTAGCCCCAAGGAGTTTGGAA
RIG-I	CGTAAGAGTGATAGAGGAATGCC	GGCTTGGGATGTGGTCTACTC
MAD5	AGGAGTCAAAGCCCACCATC	TGTTCATTCTGTGTCATGGGTT

### Statistical analyses

Continuous variables (age and ALT) are presented as mean (± SD) and viral load as mean ± Standard Error of the mean (SEM). The paired sample t-test was used for comparison of cycle threshold mean between patients and the control groups. The probability value of 0.05 or less (P ≤ 0.05) was set to know the significance level.

## Results

**Fig. 1:**
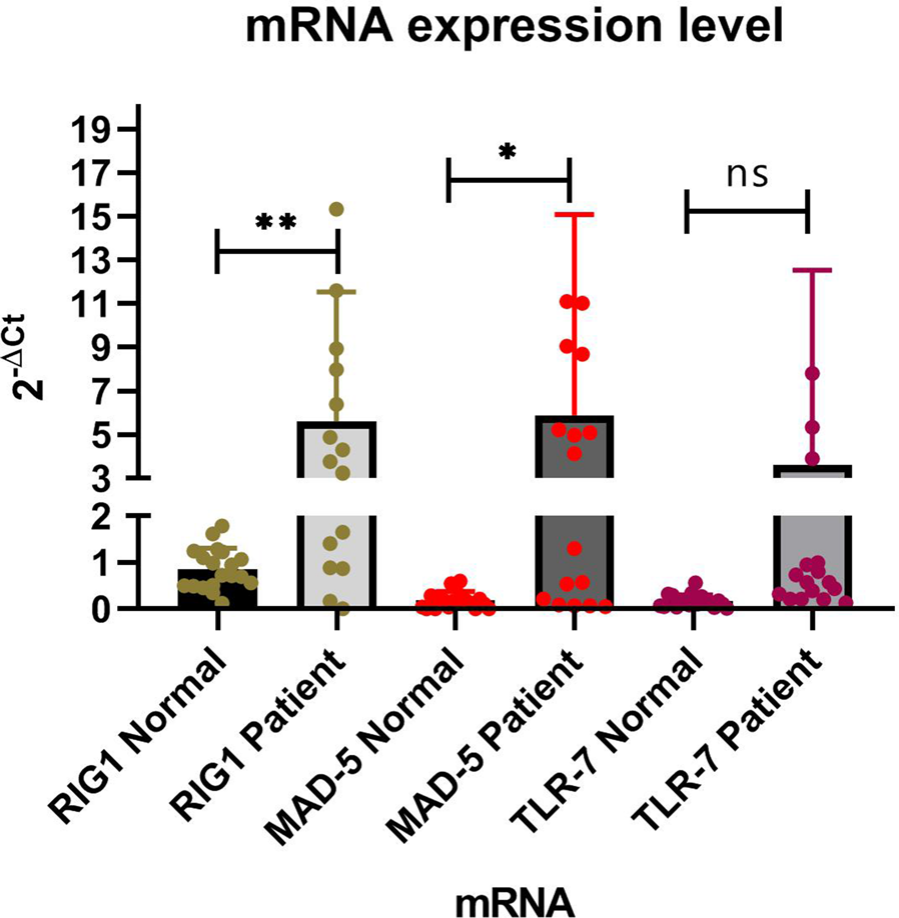
The expressions of RIG-I and MAD-5 in patients were significantly increased compared to those of controls while the increase of TLR7 expression was not statistically meaningful

40 subjects (20 HCV cases and 20 healthy people) were included in this study. There were thirteen men and seven women in each group with an average age of 44.95 years. The more information of subjects was shown in Table 2. As Fig. 1 shows, the expressions of RIG-I and MAD-5 mRNA increased more than nearby five-fold, compared with the healthy controls. The increase of of both RIG-I (P < 0.01) and MAD-5 (P < 0.05) mRNA expressions were significantly different compared with the controls. Although it appears that the amount of TLR7 mRNA increased in patients, no statistically significant changes were observed between case and healthy groups (P value:0.1).

**Table2:** Basic Demographic and viral features of the patients and healthy controls

Group genders	HCV patients	Healthy controls
Male	13	13
Female	7	7
Age mean ± SD	44.8 ± 5.7	45.1 ± 5.8
ALT/UL mean ± SD	67 ± 6.1	27 ± 1.9
Viral load copies/ml	182*10^3^

## Discussion

The activation of Host immune system against viral pathogens are initiated by the recognition of pathogen-associated molecular patterns (PAMPs) with PRRs [19, 20]. However, the expression pattern of receptors is different in viral infections, which depends on various elements including the type of virus specious, viral strategies of immune evasion and the stage of disease [13, 21, 22]. In the present study, RLRs including RIG-I and MAD-5 receptors were highly expressed in HCV positive patients while no significant changes were observed in the expression of TLR7 compared with the healthy ones. In consistent with our results, several studies have suggested that RIG-I and MAD-5 receptors would be stimulated by some conserved sequences of virus genome as a PAMP molecule, which in the following, the signaling pathways of gene expression of antiviral compounds and many cytokines, such as type I interferon would be launched to combat HCV infection [23, 24]. In contrast, in some studies, the suppression of RIG-I gene was observed in HCV positive patients [25]. The reason of this discrepancy might return to the stage of disease. To explain more, Erika et al. showed the expression of RIG-I gene was downregulated in HCV chronic patients [26]. It seems in chronic phase, NS3/NS4 serine protease of HCV directly suppresses RIG-I pathway [26, 27]. However, in initial phase of the infection, the interaction of RIG-I with HCV components strongly increase the production of type 1 INF [24]. As all patients included in our study were also new cases of HCV infection, there is a possibility that the activation of RLRs receptors can boost innate immune system in initial stage of HCV infection. According to the important role of RIG-I-like receptors in the host defense to numerous viral pathogens in preliminary stage of infection [24], the identification and synthesis of specific conserved sequences of HCV virus genome that are agonist of RLRs receptors might hopefully be applied as a vaccine adjuvant or an potential treatment option to evoke interferon signaling against HCV infection.

TLR7 has also known as one of the critical PRRs in recognition of single stranded RNA viruses and stimulation of immune system in producing INF[28]. In many studies, the RNA level of TLR7 was upregulated in PBMCs in HCV-infected patients compared to controls [8, 9]. However, the data presented in our study indicated no significant increase in TLR7 mRNA expression in HCV-infected samples. This controversy may demonstrate that an HCV-infected host is unable to respond to invading pathogens via the TLR7 signaling pathway. Because the genetic variation of TLR7 varies among people, some evidence reported that TLRs gene polymorphisms may have beneficial or deleterious effects on HBV and HCV infections and that some SNPs may influence disease progression or prognosis [29, 30]. In fact, the type of dominant polymorphism of TLR7 affects the disease state by altering gene expression or protein synthesis [31]; however, the mechanism of action is not clearly understood.

Furthermore, in some studies the expression of TLR7 gene were declined in HCV patients. For instance, Atencia et al. showed that the gene expression of TLR7 decreases in cirrhosis patients [11]. In another study, the decrease of TLR7 expression in HCV patients with hepatocarcinoma was observed [14]. Motavaf et al. also reported a significant link between the suppression of TLR7 and developing of HCV chronic infection [10]. All mentioned studies can show the lack or decline of TLR7 expression has an important role in establishing a chronic infection that ultimately ends in cirrhosis and hepatocellular carcinoma. These findings can introduce the TLR7 expression as a prognostic biomarker about a likely cancer outcome among HCV positive patients.

Recently, a few experimental studies have recommended that synthetic TLRs activators can induce potent antiviral activity against HCV [17]. However, due to the lack of TLR expression in some people, the TLR activator may be ineffective as an immune stimulant in them.

To conclude it seems in primary phase of HCV infection, RIG-I like receptors are intensely stimulated more than TLR7 in HCV patients. The lack of TLR7 gene expression might reflect the defect of host in stimulation of innate immune system through TLR7 pathway. However, the reason of this disability should be more investigated.

## Data Availability

All data and alternative results were provided in the manuscript.

## References

[1] Kim CW, Chang K-M (2013). Hepatitis C virus: virology and life cycle. Clin Mol Hepatol.

[2] Hajarizadeh B, Grebely J, Dore GJ (2013). Epidemiology and natural history of HCV infection. Nat Rev Gastroenterol Hepatol.

[3] Thompson MR, Kaminski JJ, Kurt-Jones EA, Fitzgerald KA (2011). Pattern recognition receptors and the innate immune response to viral infection. Viruses.

[4] Mogensen TH, Paludan SR (2005). Reading the viral signature by Toll-like receptors and other pattern recognition receptors. J Mol Med.

[5] Fensterl V, Sen GC (2009). Interferons and viral infections. BioFactors.

[6] Wong M-T, Chen SSL (2016). Emerging roles of interferon-stimulated genes in the innate immune response to hepatitis C virus infection. Cell Mol Immunol.

[7] Kwon Y-C, Kang J-I, Hwang SB, Ahn B-Y (2013). The ribonuclease l-dependent antiviral roles of human 2′, 5′-oligoadenylate synthetase family members against hepatitis C virus. FEBS Lett.

[8] Dehghan-Manshadi M, Hadinedoushan H, Amirbaigy MK, Zare F, Eslami G, Mirghanizade-Bafghi SA (2015). Relative expression of toll-like receptors 2 and 7 mRNA in peripheral blood of patients with hepatitis C. Hepat Mon.

[9] Dolganiuc A, Garcia C, Kodys K, Szabo G (2006). Distinct Toll-like receptor expression in monocytes and T cells in chronic HCV infection. World J Gastroenterol WJG.

[10] Motavaf M, Noorbakhsh F, Alavian SM, Sharifi Z (2014). Distinct toll-like receptor 3 and 7 expression in peripheral blood mononuclear cells from patients with chronic hepatitis C infection. Hepat Mon.

[11] Atencia R, Bustamante FJ, Valdivieso A, Arrieta A, Riñón M, Prada A (2007). Differential expression of viral PAMP receptors mRNA in peripheral blood of patients with chronic hepatitis C infection. BMC Infect Dis.

[12] Mencin A, Kluwe J, Schwabe R (2009). Toll-like receptors as targets in chronic liver diseases. Gut.

[13] Sato K, Ishikawa T, Okumura A, Yamauchi T, Sato S, Ayada M (2007). Expression of Toll-like receptors in chronic hepatitis C virus infection. J Gastroenterol Hepatol.

[14] Chang S, Kodys K, Szabo G (2010). Impaired expression and function of toll-like receptor 7 in hepatitis C virus infection in human hepatoma cells. Hepatology.

[15] Coppola N, Pisaturo M, Sagnelli C, Onorato L, Sagnelli E (2015). Role of genetic polymorphisms in hepatitis C virus chronic infection. World J Clin Cases WJCC.

[16] Mifsud EJ, Tan ACL, Jackson DC (2014). TLR agonists as modulators of the innate immune response and their potential as agents against infectious disease. Front Immunol.

[17] Horsmans Y, Berg T, Desager J, Mueller T, Schott E, Fletcher SP (2005). Isatoribine, an agonist of TLR7, reduces plasma virus concentration in chronic hepatitis C infection. Hepatology.

[18] Girkin JLN, Maltby S, Bartlett NW (2022). Toll-like receptor-agonist-based therapies for respiratory viral diseases: thinking outside the cell. Eur Respir Rev.

[19] Kumar H, Kawai T, Akira S (2011). Pathogen recognition by the innate immune system. Int Rev Immunol.

[20] Delneste Y, Beauvillain C, Jeannin P (2007). Innate immunity: structure and function of TLRs. Med Sci M/S.

[21] Chen Z, Cheng Y, Xu Y, Liao J, Zhang X, Hu Y (2008). Expression profiles and function of Toll-like receptors 2 and 4 in peripheral blood mononuclear cells of chronic hepatitis B patients. Clin Immunol.

[22] Kim G-W, Imam H, Khan M, Siddiqui A (2020). N6-Methyladenosine modification of hepatitis B and C viral RNAs attenuates host innate immunity via RIG-I signaling. J Biol Chem.

[23] Saito T, Owen DM, Jiang F, Marcotrigiano J, Gale M (2008). Innate immunity induced by composition-dependent RIG-I recognition of hepatitis C virus RNA. Nature.

[24] Gack MU (2014). Mechanisms of RIG-I-like receptor activation and manipulation by viral pathogens. J Virol.

[25] Liu HM, Gale M (2010). Hepatitis C virus evasion from RIG-I-dependent hepatic innate immunity. Gastroenterol Res Pract.

[26] Eksioglu EA, Zhu H, Bayouth L, Bess J, Liu H, Nelson DR (2011). Characterization of HCV interactions with Toll-like receptors and RIG-I in liver cells. PLoS ONE.

[27] Yoneyama M, Kikuchi M, Natsukawa T, Shinobu N, Imaizumi T, Miyagishi M (2004). The RNA helicase RIG-I has an essential function in double-stranded RNA-induced innate antiviral responses. Nat Immunol.

[28] Akira S, Takeda K (2004). Toll-like receptor signalling. Nat Rev Immunol.

[29] Schott E, Witt H, Neumann K, Bergk A, Halangk J, Weich V (2008). Association of TLR7 single nucleotide polymorphisms with chronic HCV-infection and response to interferon-a-based therapy. J Viral Hepat.

[30] Askar E, Ramadori G, Mihm S (2010). Toll-like receptor 7 rs179008/Gln11Leu gene variants in chronic hepatitis C virus infection. J Med Virol.

[31] Fakhir F, Lkhider M, Badre W, Alaoui R, Meurs EF, Pineau P (2018). Genetic variations in toll-like receptors 7 and 8 modulate natural hepatitis C outcomes and liver disease progression. Liver Int.

